# BV‐FTD: How early presentation of disease contributes in making decisions of clinicians; Diagnostic accuracy during disease trajectory

**DOI:** 10.1002/alz70857_104262

**Published:** 2025-12-25

**Authors:** Zahra Amjadi, Maryam Noroozian

**Affiliations:** ^1^ Yaadmaan Institute for Brain, Cognition and Memory Studies, Tehran, Tehran, Iran (Islamic Republic of); ^2^ Tehran University of Medical Sciences (TUMS), Tehran, Tehran, Iran (Islamic Republic of); ^3^ Cognitive Neurology, Dementia and Neuropsychiatry Research Center (CNNRC), Tehran, Tehran, Iran (Islamic Republic of)

## Abstract

**Background:**

Behavioral variant frontotemporal dementia (bvFTD) is a prominent cause of early‐onset dementia, marked by profound personality and behavioral alterations. Despite established clinical criteria, accurately diagnosing bvFTD remains challenging due to overlapping presentations with psychiatric disorders or other neurodegenerative conditions. Recent research has investigated the evolution of diagnostic accuracy, the influence of early symptoms on clinical decisions, and the delays between symptom onset and definitive diagnosis. Advances in neuroimaging, artificial intelligence (AI), and clinician expertise have shown promise in addressing these challenges.

**Method:**

A literature review was conducted using PubMed, Scopus, and Web of Science, focusing on studies from 2020 to 2025. Articles exploring diagnostic pathways, misdiagnosis rates, and longitudinal assessments of bvFTD were included. The review emphasized clinical presentation timelines, diagnostic reclassification, and predictors of accuracy, including neuroimaging findings and neuropsychological assessments.

**Result:**

Multiple studies highlighted a considerable time gap—ranging from 2 to 5 years on average— with initial misdiagnoses often including depression, bipolar disorder, or Alzheimer's disease (Papma et al., 2021; Tovar‐Rios et al., 2022). Earlier behavioral symptoms such as disinhibition, apathy, and emotional blunting were critical but often misinterpreted due to their overlap with psychiatric conditions (Woolley et al., 2011). Longitudinal follow‐up and standardized criteria significantly enhanced diagnostic specificity. Moreover, neuroimaging findings (e.g., frontal lobe atrophy patterns) and detailed neuropsychological evaluations emerged as critical factors prompting diagnostic specificity (Bang et al., 2015; Vijverberg et al., 2016). Emerging tools like AI algorithms have further enhanced predictive accuracy and reduced errors (arXiv preprints, 2021). Clinician expertise, familiarity with bvFTD's subtle early signs, and repeated assessments were consistently noted as integral to reducing diagnostic error. (Rascovsky et al., 2011).

**Conclusion:**

Literature consistently indicates that bvFTD is frequently misdiagnosed at first clinical contact due to overlapping psychiatric or other neurological syndromes. Ongoing assessments and the use of standardized diagnostic frameworks improve accuracy, yet a substantial diagnostic lag persists. Early recognition of subtle behavioral indicators is crucial for timely diagnosis, optimal clinical management, and better patient outcomes. Enhanced clinician awareness and systematic, longitudinal evaluation protocols and AI technologies remain key strategies for addressing diagnostic challenges in bvFTD.

